# Efficacy and safety of tumor treating fields (TTF) combined with chemotherapy versus chemotherapy only in the treatment of glioblastoma: a systematic review and meta-analysis

**DOI:** 10.3389/fmed.2026.1812119

**Published:** 2026-05-08

**Authors:** Naibijiang Abuliezi, Yihan Sun, Jiale Zhao, Xinge Qu, Yanyan Wu, Tiantian Liao, Gulinuer Kuerban, Xiaoyi Dun, Qinfen Wu

**Affiliations:** 1 The Second Affiliated Hospital of Xinjiang Medical University, Ürümqi, Xinjiang, China; 2 The Fifth Affiliated Hospital of Xinjiang Medical University, Ürümqi, Xinjiang, China; 3 Xinjiang Key Laboratory of Neurological Disorder Research (Lab No.: XJDX1711), Ürümqi, Xinjiang, China; 4 Xinjiang Clinical Research Center for Neurological Diseases, Ürümqi, Xinjiang, China

**Keywords:** GBM, glioblastoma, meta-analysis, systematic review, tumor treating fields

## Abstract

**Background:**

This systematic review and meta-analysis aimed to evaluate the efficacy and safety of tumor treating fields (TTF) combined with chemotherapy compared with chemotherapy alone in patients with newly diagnosed and recurrent glioblastoma (GBM).

**Method:**

A systematic search was conducted in PubMed, Embase, Web of Science, Cochrane Library, and databases from their inception to 10 December 2025. Randomized controlled trials, controlled clinical trials and observational cohort studies evaluating the efficacy and safety of TTF in GBM treatment were included. Evaluating the efficacy and safety of TTF in GBM treatment were included. Data were pooled using Stata 15 software.

**Results:**

A total of 14 studies involving 2,376 patients (both newly diagnosed and recurrent GBM) were included: 7 RCTs, 4 CCTs, and 3 observational cohort studies were included. Pooled analyses demonstrated that compared with chemotherapy alone, TTF combined with chemotherapy significantly prolonged progression-free survival (PFS) (HR = 0.62, 95% CI: 0.55–0.69, *P* < 0.0001) and overall survival (OS) (HR = 0.63, 95% CI: 0.56–0.72, *P* < 0.0001) in both newly diagnosed and recurrent GBM patients. No significant difference in systemic adverse events was observed between the two groups (OR = 1.10, 95% CI: 0.79–1.53, *P* = 0.571); however, the incidence of skin irritation was significantly higher in the TTF group (OR = 12.87, 95% CI: 7.47–22.18, *P* < 0.0001). Subgroup analyses indicated that patient diagnosis type contributed to heterogeneity across studies.

**Conclusion:**

In newly diagnosed and recurrent GBM, TTF combined with chemotherapy significantly improves survival outcomes compared with chemotherapy alone, highlighting the therapeutic value and safety of the combined treatment in both patient populations.

## Introduction

Glioblastoma (GBM) is the most common and lethal primary malignant intracranial tumor in adults, with a global incidence of approximately 3.5 per 100,000 individuals (1, 2). The current standard of care for newly diagnosed GBM consists of maximal surgical resection followed by postoperative radiotherapy administered concomitantly with temozolomide (TMZ), followed by adjuvant TMZ chemotherapy, as established in the Stupp protocol (3). However, even with this multimodal treatment, the median survival of GBM patients remains only 16–17 months, and the 5-year survival rate is approximately 10% (4). Surgery and chemotherapy alone are insufficient to prevent tumor recurrence, highlighting the urgent need for more effective and safe therapeutic strategies.

Alternating electric fields have demonstrated a wide spectrum of biological effects. Specifically, intermediate-frequency (100–300 kHz), low-intensity alternating electric fields can disrupt tumor cell division and proliferation while also exhibiting potential chemosensitizing effects (5). Importantly, these fields are associated with favorable safety profiles and minimal systemic toxicity (6). Tumor treating fields (TTF), developed on the basis of these bioelectrical effects, represent a novel physical therapeutic modality and offer a promising new option for patients with glioblastoma (7).

Previous small-scale, single-arm clinical studies evaluated TTF combined with radiotherapy and chemotherapy for GBM, showing that this regimen is safe, feasible, and associated with limited toxicity (8). In newly diagnosed glioblastoma, a phase III clinical trial (9) also reported favorable outcomes. In this trial, 695 patients who had undergone standard surgery and concurrent chemoradiotherapy were randomly assigned in a 2:1 ratio to receive either TTF combined with temozolomide or temozolomide alone. The results showed that the addition of TTF significantly prolonged both overall survival (OS) and progression-free survival (PFS).

However, not all clinical studies confirmed its efficacy in enhancing chemotherapy outcomes. For example, in a clinical controlled trial conducted by She et al. (10), the addition of TTF to chemotherapy did not significantly improve OS in patients with either newly diagnosed or recurrent GBM. Given these inconsistent findings, a meta-analysis provides a valuable approach to systematically evaluate the efficacy and safety of TTF in newly diagnosed and recurrent GBM.

## Materials and methods

This systematic review and meta-analysis was conducted in accordance with the Preferred Reporting Items for Systematic Reviews and Meta-Analyses (PRISMA) 2020 guidelines.

### Eligibility criteria

The inclusion criteria: (1) Research type: study type should be either clinical controlled trial (RCT), quasi-controlled trial or observational cohort study. (2) Research subjects: studies should enroll patients diagnosed with GBM (including newly diagnosed patients and recurrent patients). (3) Intervention: studies should include an experimental group receiving TTF + chemotherapy and a control group receiving only chemotherapy. Temozolomide was used as the first-line chemotherapy regimen, or second-line chemotherapy drugs could be used. (4) Control process: studies are required to describe the process of selecting, grouping, and controlling the trial. (5) Outcomes: studies should report outcomes after intervention, such as PFS, OS, and incidence of adverse reactions.

The exclusion criteria: (1) The enrolled subjects are non-GBM populations; Research on animals will also be excluded; (2) Only one of the duplicate studies with complete data records will be retained, and the rest will be excluded; (3) Research of investigation, case analysis, review, observational studies, and other types will be excluded; (4) Studies without sample size, baseline data, follow-up records, and outcome data will be excluded.

### Databases and search strategy

Online databases Pubmed, Embase, Web of Science, and Cochrane Library were used as the data sources for this study. The publication deadline for the literature is before 10 December 2025. We also searched for unpublished clinical studies on Clinicaltrials.org. Search keywords were: “Tumor treating fields,” “TTF,” “Temozolomide,” “TMZ,” “Chemotherapy,” “glioma tumor,” “Glioblastoma.” The free-style keyword search and advance search were applied to retrieve studies.

### Screening process

After the studies were retrieved and duplicates were removed, two reviewers independently reviewed the abstract and full text of the studies based on the defined criteria to check their eligibility.

### Risk of bias

The risk of bias of the included randomized controlled trials was assessed using the Cochrane Risk of Bias tool, version 2.0 (RoB 2) (11). This tool evaluates methodological quality across five domains: (1) bias arising from the randomization process, (2) bias due to deviations from intended interventions, (3) bias due to missing outcome data, (4) bias in measurement of the outcome, and (5) bias in selection of the reported result. Each domain was judged as “low risk,” “some concerns,” or “high risk” according to the signaling questions and algorithm provided in the RoB 2 guidance. An overall risk-of-bias judgment for each study was derived based on domain-level assessments following Cochrane recommendations. For non-randomized controlled trials (CCTs) and observational cohort studies, risk of bias was evaluated using the ROBINS-I tool (Risk Of Bias In Non-randomized Studies–of Interventions) (12). Two reviewers independently conducted the assessment, and discrepancies were resolved through discussion or consultation with a third reviewer when necessary.

### Data extraction

Data were independently extracted by two reviewers using a predefined standardized data extraction form. Extracted information included study characteristics (first author and publication year), participant characteristics (mean age, sex distribution, and diagnosis type), intervention details (treatment strategies and group allocation), methodological information (sample size and follow-up duration), and outcome measures, particularly survival-related endpoints. Any discrepancies were resolved through discussion or consultation with a third reviewer to ensure accuracy and consistency.

### Statistical analysis

The primary outcomes were progression-free survival (PFS) and overall survival (OS), which were synthesized using hazard ratios (HRs) with corresponding 95% confidence intervals (CIs) as the standard effect measures for time-to-event data. Log-transformed HRs and their standard errors were pooled for analysis; when HRs were not directly reported, they were extracted from available statistics or reconstructed from Kaplan–Meier curves using established methods. For safety outcomes, including systemic adverse events and device-related skin irritation, odds ratios (ORs) with 95% CIs were calculated. Random-effects models based on the DerSimonian–Laird estimator were applied as the primary analytical approach to account for expected clinical and methodological heterogeneity, while fixed-effect models were explored for comparison where appropriate. Statistical heterogeneity was assessed using Cochran’s Q test and quantified with the I^2^ statistic, with values of approximately 25, 50, and 75% interpreted as low, moderate, and high heterogeneity, respectively. Potential sources of heterogeneity were investigated through predefined subgroup analyses according to diagnostic status and study characteristics. Sensitivity analyses were conducted using influence diagnostics and leave-one-out procedures implemented through the “InfluenceAnalysis” function in the dmetar package to evaluate the robustness of pooled estimates. Publication bias was assessed by visual inspection of funnel plots and further examined using Egger’s regression test when sufficient studies were available. All statistical analyses were performed using the meta and dmetar packages in R software (version 4.4.1), and statistical significance was defined as a two-sided *P*-value < 0.05.

## Results

### The selection flow

**FIGURE 1:**
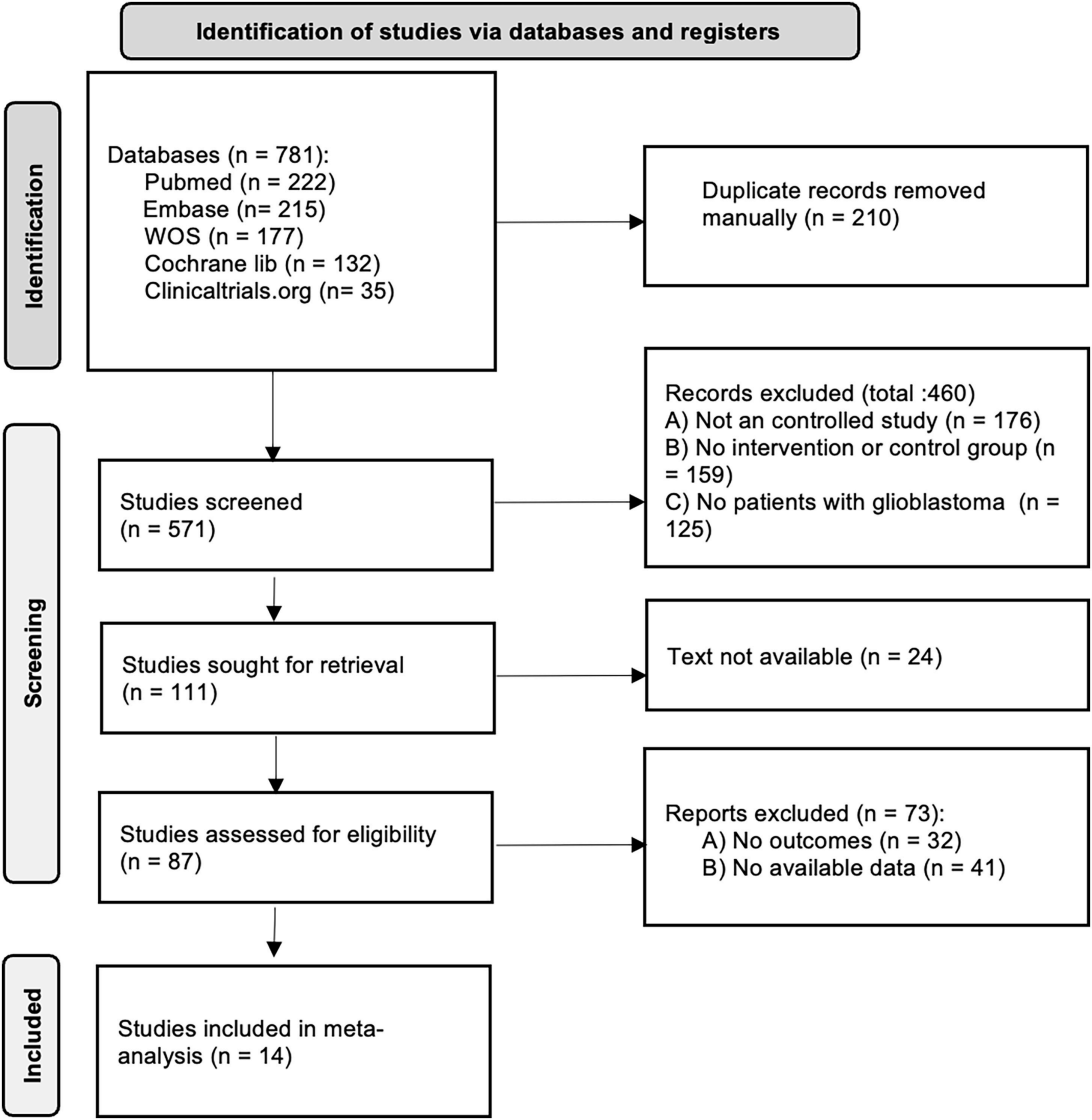
Study selection flow chart.

Figure 1 shows the literature selection process. Initially, 781 articles were retrieved, and after deduplication and screening, 14 articles (9, 10, 13–24) were finally included for quantitative analysis.

### The characteristics of studies

The basic characteristics, intervention measures, and outcome indicators of all literature are listed in Table 1. A total of 14 studies were included, with 7 RCTs, 4 controlled trials and 3 observational cohort studies. These studies were published from 2015 to 2025, with sample sizes ranging from 38 to 695. A total of 11 studies used TMZ as the chemotherapy regimen, and 3 article used second-line chemotherapy drugs as the chemotherapy regimen. A total of 2,376 patients were enrolled, with 1,289 of them in the intervention group and 1,087 of them in the control group.

**TABLE 1:** Basic characteristics, grouping, and intervention methods of the studies.

Study	Design	Age (years)	Sex (male)	Sample size (E/C)	Diagnosis	Intervention	Control	Follow-up (mo)
Stupp et al. (9)	RCT	55.8 ± 11.1	458 (66%)	695 (466/229)	NdGBM	TTFields + TMZ	TMZ	38 (18, 60)
She et al. (10)	RCT	54 (33–63)	53 (57%)	52 (13/39)	NdGBM	TTFields + TMZ	TMZ	NR
She et al. (10)	RCT	54 (33–63)	53 (57%)	41 (13/28)	RGBM	TTFields + TMZ	TMZ	NR
Vymazal et al. (13)	RCT	47.3 ± 11.8	55 (55.1%)	109 (55/54)	NdGBM	TTFields + TMZ	TMZ	NR
Kesari and Ram (14)	RCT	57 (29–83)	153 (75%)	204 (144/60)	RGBM	TTFields + 2nd line chemotherapy	2nd line chemotherapy	12.6
Kim et al. (15)	RCT	52.1	26 (66.7%)	38 (24/14)	NdGBM	TTFields + TMZ	TMZ	NR
Chen et al. (16)	CCTs	49.98 ± 13.40	162 (60.2%)	267 (63/204)	NdGBM	TTFields + TMZ	TMZ	NR
Liu et al. (17)	CCTs	61 (28, 81)	61 (58.6%)	104 (37/67)	NdGBM	TTFields + TMZ	TMZ	NR
Pandey et al. (18)	RCT	59 (26, 79)	40 (35.7%)	112 (55/57)	NdGBM	TTFields + TMZ	TMZ	NR
Ballo et al. (19)	CCTs	60 (34, 87)	62 (68%)	91 (59/32)	NdGBM	TTFields + TMZ	TMZ	NR
Onken et al. (20)	Observational cohort study	50 (36, 64)	39 (68.4%)	57 (30/27)	NdGBM	TTFields + TMZ	TMZ	NR
Wong et al. (21)	Observational cohort study	57 (30, 77)	23 (62.2%)	37 (3/34)	RGBM	TTFields + 2nd line chemotherapy	2nd line chemotherapy	NR
Zhang et al. (22)	Observational cohort study	52.15 ± 10.89	32 (61.5%)	52 (26/26)	NdGBM	TTFields + TMZ	TMZ	10.6 (9.57–11.63)
Riegel et al. (23)	CCTs	60 (27–86)	118 (56.7%)	208 (109/99)	NdGBM	TTFields + TMZ	TMZ	13.1 (1.6–93.8)
Zhu et al. (24)	RCT	57.0 (23–80)	198 (64.1%)	309 (192/117)	RGBM	TTFields + 2nd line chemotherapy	2nd line chemotherapy	12

NR, not reported; RCT, randomized controlled trial; CCTs, controlled clinical trial; E/C, experiment/control; TMZ, temozolomide; GBM, glioblastoma; NdGBM, newly diagnosed GBM; RGBM, recurrent GBM.

### Assessment of risk of bias

**FIGURE 2:**
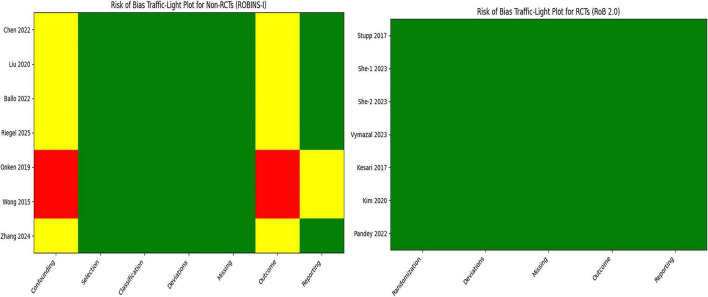
The overall assessment of risk of bias.

The methodological quality of the included studies was evaluated according to study design. Randomized controlled trials (RCTs, *n* = 7) were assessed using the Cochrane RoB 2.0 tool, which evaluates five domains: randomization process, deviations from intended interventions, missing outcome data, outcome measurement, and selective reporting. All RCTs were judged to have low risk of bias across all domains, indicating high methodological quality. Domains related to confounding, selection of participants, and classification of interventions are not applicable to RCTs (Table 2 and Figure 2).

**TABLE 2:** Bias risk assessment based on ROB 2.0.

Study	Design	Assessment tool	Randomization/ assignment	Deviations from intended interventions	Missing outcome data	Outcome measurement	Selective reporting	Confounding	Selection of participants	Classification of interventions	Overall risk
Stupp et al. (9)	RCT	RoB 2.0	Low	Low	Low	Low	Low	–	–	–	Low
She et al. (10)	RCT	RoB 2.0	Low	Low	Low	Low	Low	–	–	–	Low
She et al. (10)	RCT	RoB 2.0	Low	Low	Low	Low	Low	–	–	–	Low
Vymazal et al. (13)	RCT	RoB 2.0	Low	Low	Low	Low	Low	–	–	–	Low
Kesari and Ram (14)	RCT	RoB 2.0	Low	Low	Low	Low	Low	–	–	–	Low
Kim et al. (15)	RCT	RoB 2.0	Low	Low	Low	Low	Low	–	–	–	Low
Pandey et al. (18)	RCT	RoB 2.0	Low	Low	Low	Low	Low	–	–	–	Low
Chen et al. (16)	CCT	ROBINS-I	Moderate	Low	Low	Low	Low	Moderate	Low	Moderate	Moderate
Liu et al. (17)	CCT	ROBINS-I	Moderate	Low	Low	Low	Low	Moderate	Low	Moderate	Moderate
Ballo et al. (19)	CCT	ROBINS-I	Moderate	Low	Low	Low	Low	Moderate	Low	Moderate	Moderate
Riegel et al. (23)	CCT	ROBINS-I	Moderate	Low	Low	Low	Low	Moderate	Low	Moderate	Moderate
Onken et al. (20)	Observational	ROBINS-I	Serious	Low	Low	Low	Low	Serious	Moderate	Serious	Serious
Wong et al. (21)	Observational	ROBINS-I	Serious	Low	Low	Low	Low	Serious	Moderate	Serious	Serious
Zhang et al. (22)	Observational	ROBINS-I	Moderate	Low	Low	Low	Low	Moderate	Low	Moderate	Moderate

For the non-randomized studies (CCTs and observational cohort studies, *n* = 7), the ROBINS-I tool was applied, assessing seven domains: confounding, selection of participants, classification of interventions, deviations from intended interventions, missing outcome data, outcome measurement, and selective reporting. Among these studies, confounding was the most frequent source of bias, with four studies rated as moderate and two studies as serious risk. Deviations from intended interventions, missing outcome data, and outcome measurement were generally rated low. Some studies exhibited moderate to serious risk in domains related to selection of participants and classification of interventions. Overall, the non-randomized studies were considered to have moderate to serious risk of bias, reflecting the inherent limitations of non-randomized designs (Table 2 and Figure 2).

### Pooled effect sizes

#### TTF’s HR values for PFS and OS

**FIGURE 3:**
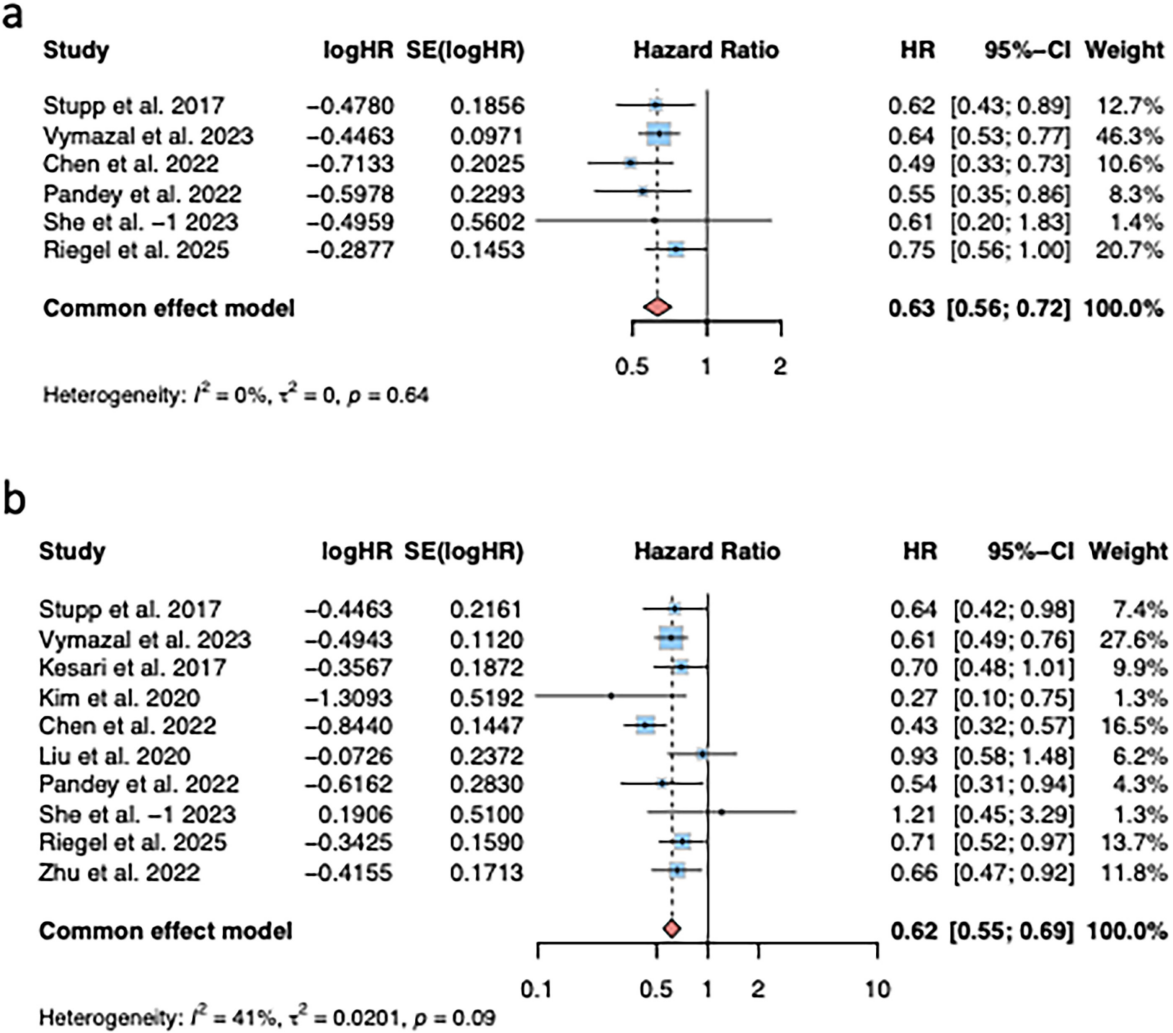
Pooled hazard ratios (HR) for the effect of TTF on survival outcomes in patients with glioblastoma. **(a)** Progression-free survival (PFS); **(b)** overall survival (OS).

Six trials reported the HR values of TTF relative to PFS, and the pooled HR was: (HR = 0.63, 95% CI (0.56; 0.72), *P* < 0.0001) (Figure 3A); Ten trials reported the HR values of TTF relative to OS, and the pooled HR was: (HR = 0.62, 95% CI (0.55; 0.69), *P* < 0.0001) (Figure 3B).

#### Adverse reaction incidence rate

**FIGURE 4:**
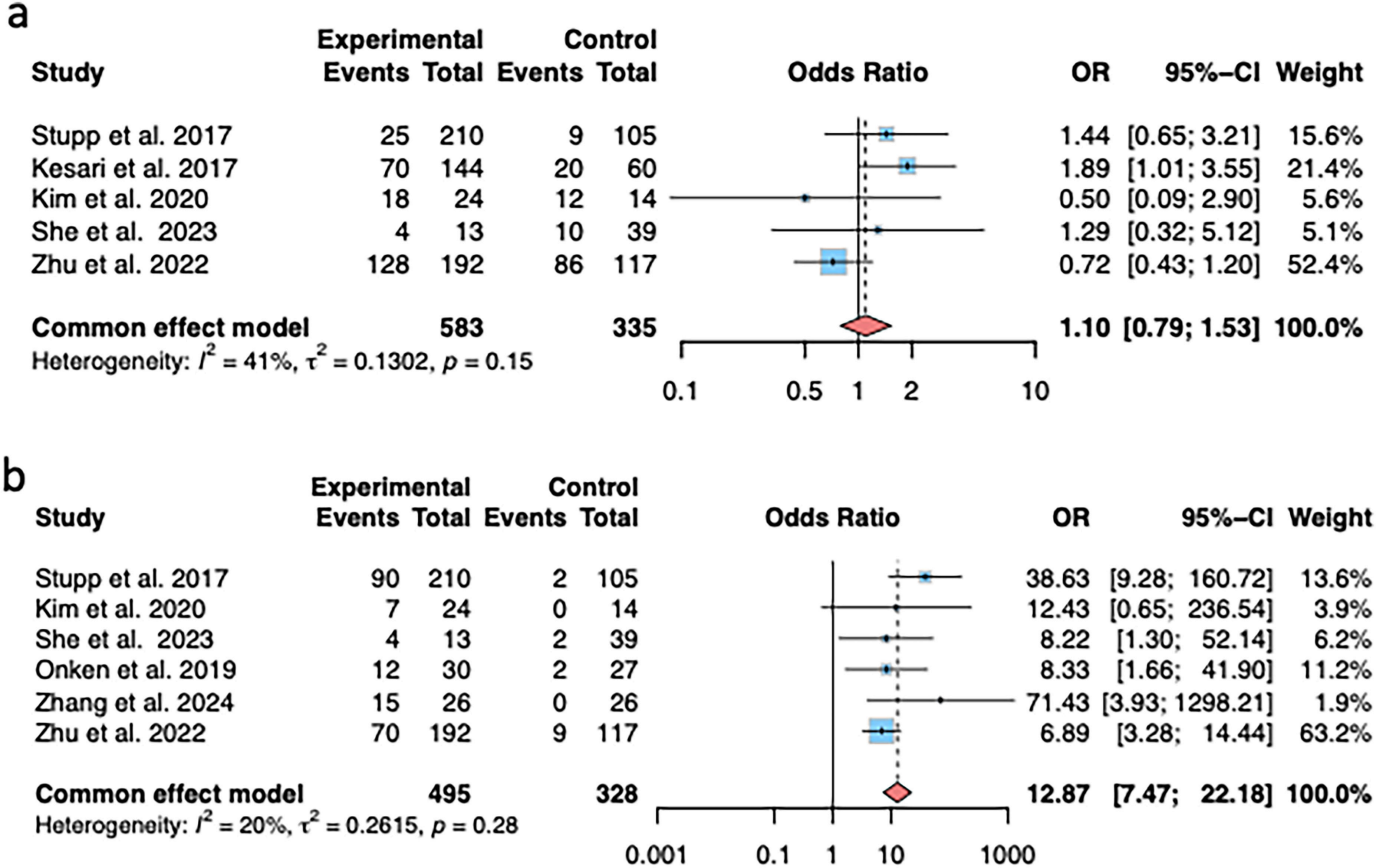
Comparison of post-treatment adverse reactions between the intervention and control groups. **(a)** Incidence of systemic adverse reactions; **(b)** Incidence of skin irritation.

Five articles reported the incidence of systemic adverse reactions, no significant difference was found between the intervention and the control groups (OR = 1.10, 95% CI (0.79; 1.53), *P* = 0.571) (Figure 4A). Six articles reported on the incidence of skin irritation adverse reactions, significant difference was found between the two groups (OR = 12.87, 95% CI (7.47; 22.18), *P* < 0.0001) (Figure 4B).

#### Subgroup analysis

**FIGURE 5:**
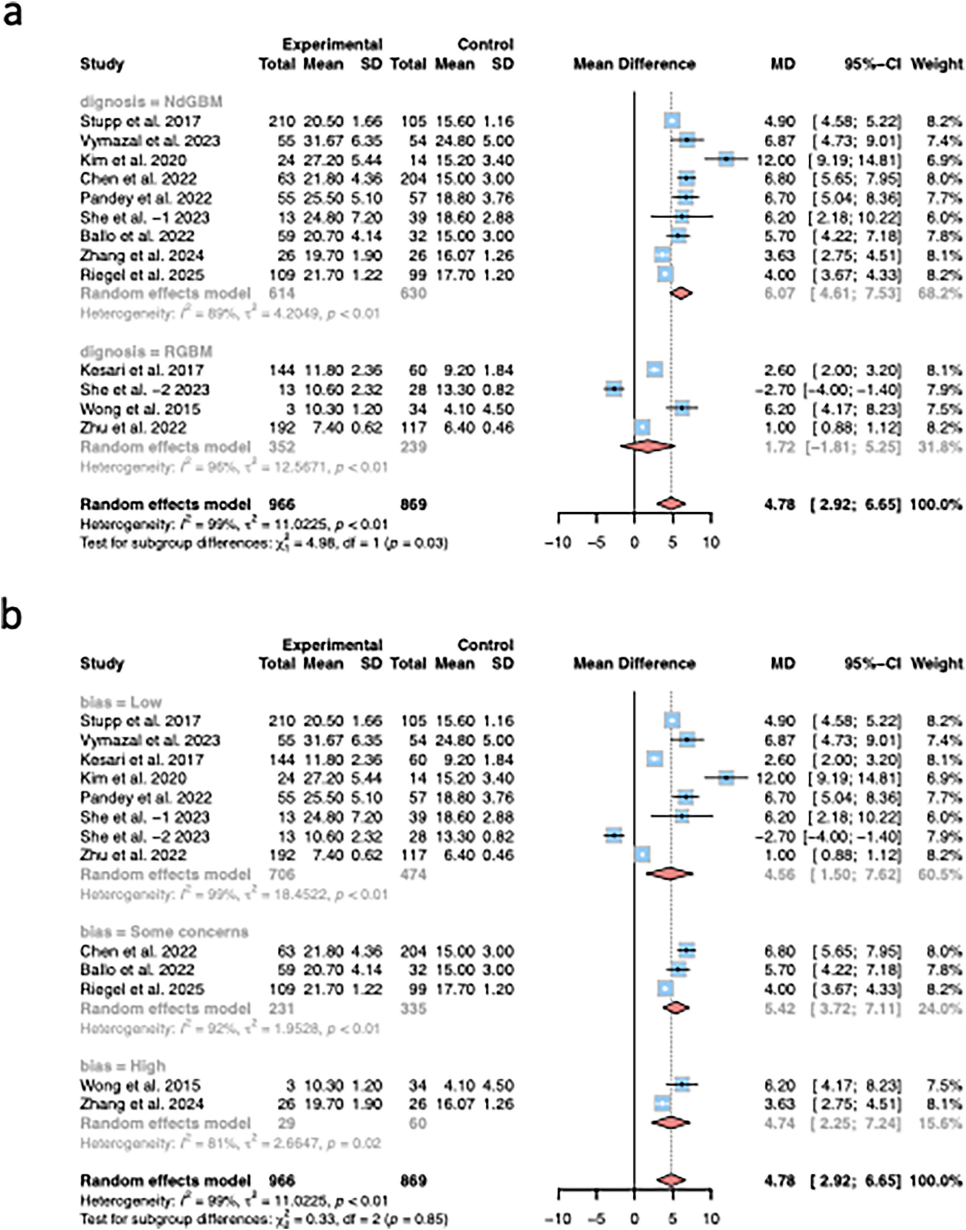
Comparison of post-treatment adverse reactions between the intervention and the control group. **(a)** The incidence of systemic adverse reactions throughout the body; **(b)** The incidence of skin irritation.

Due to significant heterogeneity observed during OS pooling, the studies were sub-grouped into two groups based on the diagnostic type of patients (NdGBM, RGBM) (Figure 5A). Significant difference was found between subgroups (*P* = 0.01), indicating that the diagnostic type was one kind of source for heterogeneity. The studies were sub-grouped into three groups based on the risk of bias (low, some concerns, high) (Figure 5B). No significant difference was found between subgroups (*P* = 0.85), indicating that the risk of bias was not one kind of source for heterogeneity.

#### Publication bias

**FIGURE 6:**
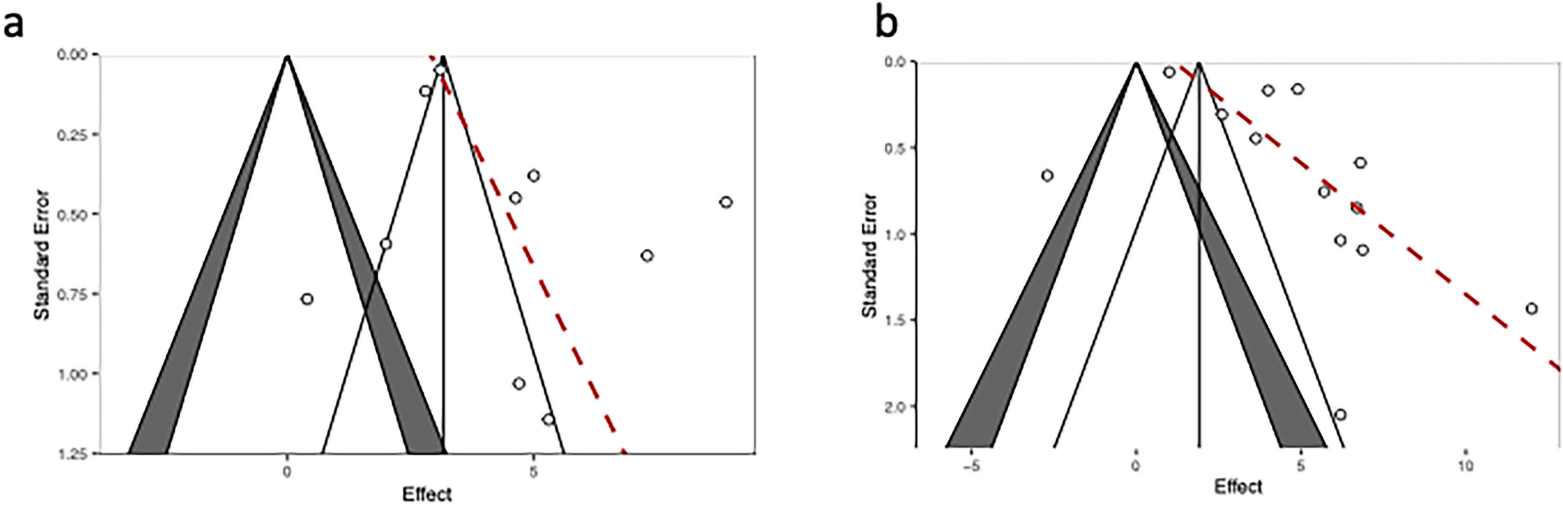
Assessment of publication bias. **(a)** Funnel plot for PFS; **(b)** Funnel plot for OS. The red dashed line represents the regression line from Egger’s test.

The funnel plots for PFS and OS were shown in Figure 6. The egger’s test result for PFS was *t* = 1.609, *P* = 0.146, indicating minimum publication bias (Figure 6A). The egger’s test result for OS was *t* = 2.398, *P* = 0.035, indicating significant publication bias (Figure 6B).

## Discussion

Tumor treating fields (TTF) represent an emerging therapeutic modality for cancer and have demonstrated promising efficacy in pancreatic cancer (25), osteosarcoma (26), and non-small cell lung cancer (27). The device is composed of three components: transducers, connecting wires, and a battery. By placing transducers at targeted anatomical sites, TTF generates medium-frequency, low-intensity alternating electric fields that disrupt the mitotic process of tumor cells (28). The underlying mechanism involves inhibition of microtubule polymerization and induction of organelle electrophoresis, which interfere with intracellular structures and ultimately suppress tumor cell migration (29). Owing to its portability, favorable safety profile, and clinical effectiveness, TTF has been approved in several countries as a treatment option for cancer.

Kirson et al. (30) reported that TTF combined with TMZ enhances chemosensitivity through synergistic anti-tumor effects, supporting the feasibility of this combination therapy. In the present analysis, nine controlled studies were included to evaluate the efficacy and safety of TTF in combination with chemotherapy compared with chemotherapy alone for the treatment of glioblastoma. Of these, eight studies employed TMZ as the first-line chemotherapeutic agent, while one study by Kesari and Ram (14) investigated the use of a second-line chemotherapy regimen in recurrent glioblastoma. The pooled results demonstrated that TTF combined with chemotherapy (either first- or second-line) significantly improved both PFS and OS compared with chemotherapy alone. Moreover, TTF emerged as an independent protective factor influencing both PFS and OS, further substantiating the synergistic effect between TTF and TMZ. Similarly, Regev et al. (31) conducted a comprehensive single-arm clinical study and reported satisfactory clinical efficacy of TTF in glioblastoma. However, the single-arm design limited direct comparison with a control group. By including only controlled clinical trials, the present study provides more robust evidence supporting the potential benefit of TTF combined with TMZ over chemotherapy alone.

TTF not only exerts direct anti-tumor effects but also enhances chemosensitivity. At a frequency of 150 kHz and an intensity of 1.5 V/cm, TTF combined with paclitaxel significantly inhibits the proliferation of breast cancer cells, likely due to their shared anti-microtubule mechanism of action. Furthermore, the combination of TTF with other chemotherapeutic agents demonstrates additive or synergistic effects (32). Importantly, TTF has been shown to reverse tumor drug resistance, with comparable efficacy observed in resistant and non-resistant cancer cell strains. This effect is hypothesized to be associated with disruption of cytoskeletal integrity, including microtubules and mitochondria, while the reversal of resistance may be mediated through inhibition of microtubule formation and induction of dielectric electrophoresis (33).

Scherm et al. (34) investigated multiple therapeutic agents including bevacizumab for newly diagnosed GBM and found that neither of these agents demonstrated a significant improvement in OS. In contrast, the pooled hazard ratios in the present study indicated that TTF significantly improved both PFS (HR = 0.63, *P* < 0.0001) and OS (HR = 0.62, *P* < 0.0001). These findings suggest that, as a non-pharmacological intervention, TTF may achieve superior survival benefits compared with certain immunotherapeutic agents in the treatment of glioblastoma.

In this study, substantial heterogeneity was observed across the nine included studies. Subgroup analysis by disease type—NDGBM versus RGBM—revealed significant differences between subgroups, suggesting that patient diagnostic characteristics represent a major source of heterogeneity. Notably, TTF did not demonstrate significant improvements in PFS or OS within the RGBM subgroup, indicating that its clinical utility in recurrent disease may be limited. However, as this subgroup included only two studies, the strength of evidence remains low. Moreover, given the wide variability in baseline characteristics of recurrent GBM, including age, Karnofsky Performance Status (KPS), and frequency of recurrence, the therapeutic role of TTF in this setting may differ considerably. Further well-designed studies are therefore warranted to clarify the potential benefit of TTF in combination with chemotherapy for recurrent GBM.

Regarding safety, four studies reported systemic adverse events associated with TTF, and the pooled analysis indicated no significant increase in systemic toxicities. However, Stupp et al. (9) reported that approximately 43% of patients receiving TTF plus TMZ developed moderate-to-severe skin irritation, compared with only 2% in the TMZ-only group. Similar findings were reported by Kim et al. (15) and She et al. (10). Pooled results from three studies demonstrated a markedly higher risk of skin irritations with TTF (OR = 12.87, *P* < 0.0001). Skin irritation represents the most common adverse event of TTF, primarily resulting from prolonged electrode–skin contact, which can progress to irritant or allergic contact dermatitis, erosion, ulceration, secondary infection, or pustule formation (35). Nevertheless, these dermatologic toxicities are generally manageable. Preventive strategies include regular scalp care, periodic adjustment of electrode placement to minimize continuous contact with the same skin area, and early intervention with topical corticosteroids or antibiotics when necessary (36).

In the study by Taphoorn et al. (37), quality of life (QoL) outcomes were compared between patients receiving TTF plus TMZ and those receiving TMZ alone. The TTF + TMZ group demonstrated superior outcomes in overall health, physical functioning, emotional wellbeing, pain reduction, and mobility, highlighting the potential of TTF to improve QoL in GBM patients. In contrast, Bernard et al. (38) evaluated TTF from a health economics perspective and reported that, despite prolonging survival, TTF is associated with high costs and limited cost-effectiveness, emphasizing the need for policy-level interventions to improve accessibility and sustainability. Future research should focus not only on elucidating the mechanisms of TTF but also on refining its economic value to optimize personalized treatment strategies for gliomas.

Several mechanistic studies have demonstrated that intermediate-frequency, low-intensity alternating electric fields can disrupt tumor cell division, inhibit mitotic spindle formation, and enhance chemosensitivity in glioblastoma cell lines (39). While these findings provide a plausible biological rationale for TTF, their direct relevance to clinical outcomes requires careful interpretation. For example, although *in vitro* studies suggest that TTF may increase tumor cell apoptosis, clinical trials indicate that the magnitude of PFS and OS benefits is moderate. Differences in tumor heterogeneity, microenvironment, and patient compliance likely modulate the translation of these mechanisms into measurable clinical effects. By integrating mechanistic insights with trial data, we can better understand how TTF contributes to survival outcomes and identify potential factors influencing treatment efficacy.

In sensitivity analyses, the robustness of the results was confirmed. Nonetheless, several limitations remain. (a) While this study affirmed the therapeutic role of TTF combined with chemotherapy in GBM, the underlying mechanisms require further clarification. (b) Literature on recurrent GBM is limited, and the efficacy of TTF in this setting cannot yet be determined. (c) For some outcomes, such as adverse event reporting, the number of studies was small, resulting in a low level of evidence. (d) During data consolidation, a significant publication bias was identified, suggesting that existing studies are more likely to report favorable outcomes of TTF.

## Conclusion

The efficacy of TTF combined with chemotherapy is significantly better than chemotherapy alone both for newly diagnosed and recurrent glioblastoma, and it is relatively safe; However, for recurrent GBM, the level of evidence for the therapeutic benefits brought by TTF is relatively low and further confirmation is still needed.

## Data Availability

The original contributions presented in this study are included in the article/supplementary material, further inquiries can be directed to the corresponding author.
